# Do Journals Have Preferences? Insights from *The Journal of Higher Education*

**DOI:** 10.1007/s10755-022-09634-5

**Published:** 2022-10-28

**Authors:** Nicholas Havey, Mitchell J. Chang

**Affiliations:** University of California, Los Angeles, CA USA

**Keywords:** Publishing, Journals, Quantitative methods

## Abstract

Using five years of publishing data from the Journal of Higher Education, we describe the publication pipeline at the journal, explore trends with respect to topic, the geographic distribution of authors, and each paper's methodological approach. Following the presentation of these trends, we discuss implications for the field of higher education and those publishing within it.

Publishing intersects with the three main duties for most tenure-track faculty: teaching, service, and research. With respect to teaching, syllabi are constructed from the published annals of each respective field and students’ term papers are, hopefully, filled with variably-formatted reference to published scholarship. When it comes to service, there is an expectation that academics engage in and benefit from peer review and that, when they have advanced to a certain stage in their career, they voluntarily serve as editors for journals who rely on their expertise and labor. And of course, research, the scholarly pursuit and production of new knowledge that is at the core of most academics’ interests and their CVs, inherently relies upon publishing, and the labor that makes it happen. Publishing is thus a crucial and necessary part of academic life (Wilkins & Huisman, 2015). The adage “publish or perish” is an adage for a reason and our study seeks to contribute to the ongoing effort to demystify and explain the publication processes, its intricacies, and the patterns and trends associated with the study of higher education.

Much of the scholarship concerning academic publishing is field specific and disciplinary in nature (Williams et al., 2018). Indeed, empirical research on publishing must also go through a peer review process that tends to lean in favor of the interests, norms, and trends of the field that are inhabited by the reviewers. Given this proclivity, it makes sense that refereed peer-reviewed journals would exhibit certain preferences. With respect to the field of higher education, several studies have examined the methodological and gender distribution of published research (Hutchinson & Lovell, 2004; Johnson et al., 2016; Wells et al., 2015; Williams et al., 2018), the geographic diversity of contributors to higher education research journals (Fitzgerald & Jiang, 2020; Mason et al., 2021), and the impact and influence of those journals on future research (Earp, 2010; Wilkins & Huisman, 2015).

For example, Hutchinson and Lovell (2004) explored the methodological characteristics of research published in “key journals in higher education” and found that quantitative studies dominated journal publications. The dominance of this methodological approach led them to suggest enhancing training of doctoral students by increasing methodological course requirements. A decade later, this study was replicated by Wells and colleagues (2015) who used data representing the same journals but with a more recent time period. They too found that published higher education research was dominated by quantitative approaches. They similarly called for changes in doctoral education but also called for changes to the field, specifically the consideration, acceptance, and subsequent publication of non-quantitative studies and theoretical works.

More recently, Johnson and colleagues (2016) analyzed the top journals of research on higher education as identified by impact factors and indexing score. Similar to previous studies, they found that 60% of all published work in those journals utilized quantitative methodologies. Additionally, they reported that in a sample of 587 published papers over four years, 29% of contributors were affiliated with the same five academic institutions. So, not only were quantitative studies overrepresented in the top journals but so were faculty researchers with appointments at a select group of institutions.

The dominance of quantitative methodologies in empirical journals that focus on higher education was confirmed yet again by Williams and colleagues (2018). They also reported gender differences in methodological approach, with women employing qualitative methodologies far more often than men. Other studies that have examined publication trends in higher education journals have found that the authors of the publications are largely based in the United States and reside in a handful of academic institutions (Fitzgerald & Jiang, 2020; Mason et al., 2021). In sum, the existing research suggests that when it comes to publishing in research journals that cover higher education, there is a preference for quantitative methodologies, which tend to be authored by those who reside in a small group of institutions in the United States.

Since publishing is central to academic life (Wilkins & Huisman, 2015), examining the related processes and outcomes can provide unique insights into trends within a field of study. Although previous studies about publishing have been illuminating, a limitation that they share is that they have all focused on published work that had survived the peer review process, been accepted for publication in a journal, gone through a production process that likely included copy-editing, reference-checking, and formatting, and was subsequently made available for public consumption. Absent from their analyses are the hundreds, or thousands, depending on the journal, of submissions that never made it through this process. Since those studies focus only on the end product, they do not reveal insights into the full publication process. For example, journals typically keep track of internal metrics and logistics concerning manuscript processing time, acceptance rates, and peer reviewers. Potential contributors, especially emerging scholars, might benefit from those additional insights, helping them to understand better the inner workings of academic publishing so that they can make a more informed decision about both how and where to publish their research.

Accordingly, this study draws from our insights as former Chief and Managing Editors of *The Journal of Higher Education* (JHE). This journal was established in 1930 and is considered by reputation and by a host of metrics to be one of the top research journals covering higher education. It is currently owned by The Ohio State University Press. Although the review process that we describe and the data that we analyzed are limited to JHE, our findings still contribute to the existing knowledge base about publishing in higher education journals in two key ways: 1) by presenting metrics at each stage of the publication timeline for manuscripts submitted to JHE within a recent five-year period and 2) by analyzing all submitted manuscripts within this period to explore methodological, geographic, and topical trends. Following the presentation of the publication timeline and metrics, we further analyze our sample of manuscripts to address the following research questions:

1. Is there a preference for manuscripts that utilize quantitative methodologies*?*
2. Is there a preference for manuscripts submitted by authors who reside in the United States?
3. Is there a preference for manuscripts that address certain topical areas?

## Publishing Process and Trends for *The Journal of Higher Education*

The publication process at *The Journal of Higher Education* starts at submission. After reviewing the journal’s instructions for authors, scope and content, and recently published work, interested authors submit their manuscripts for consideration through an online submission portal, *Editorial Manager*. Between January 1^st^, 2017 and February 16^th^, 2022, the timespan that overlaps with one of the co-author’s tenure as the Editor in Chief of *The Journal of Higher Education,* the journal received 4,167 unique manuscript submissions. This sample of manuscripts serves as the core data for our analyses.

### Technical Check

Following submission, each manuscript enters editorial processing. At this stage, the editorial assistant and the managing editor of the journal review the manuscript for the first time and second time respectively, assessing its relevance to the journal (i.e., is it a topic central to higher education scholarship?), whether there are any technical issues (did the author(s) follow the instructions posted on the journal website?), and make a preliminary decision. At this stage, the manuscript generally follows three routes: 1) it is returned to the author to correct technical issues (it exceeds the word limit, it is not formatted to journal style, it includes identifying information, or it shares concerning similarities with other published work that may suggest plagiarism, or more commonly that the submission is the product of a larger work such as a dissertation or has been published as a preprint or working paper by the authors, etc.), 2) the manuscript is desk rejected due to improper fit with the journal (i.e., it is a manuscript about an innovative change to dental education and not necessarily about the field of higher education, for example), or 3) is forwarded to either the Editor in Chief or one of the Associate Editors for secondary review. Between 2017 and 2022 we returned 46 manuscripts to the author for technical issues that resulted in the author withdrawing the manuscript from consideration; hundreds of other manuscripts were returned to the author for technical issues and resubmitted to the journal. All other manuscripts (4,121) were processed and either rejected or assigned to an editor. The average time from submission to editor assignment was 2.8 days, the average time from submission to reviewer invitations was 30 days, and the average time from submission to first decision was 27.8 days (this number reflects the high volume of manuscripts that *The Journal of Higher Education* rejects at the desk level and after secondary editorial review, detailed below).

### Peer Review

Once manuscripts have exited the technical check and been assigned to the editors and not rejected at that level, they enter the peer review stage. At this stage, between 2 and 8 reviewers are invited by editors to review the paper. Sometimes this stage is drawn out because reviewers decline invitations due to a number of reasons, including but not limited to having other commitments, a lack of methodological or topical expertise, and other personal and professional responsibilities. Between 2017 and 2022 we invited 2,629 reviewers, 451 of whom declined to review the manuscripts they were invited for, 1,860 of whom completed reviews, and 47 of whom are still completing their reviews. The average number of reviewers invited per manuscript was four, reflecting the reality that many invited reviewers declined to review. During this period, 166 reviewers were uninvited as reviewers and 105 were terminated as reviewers because of failure to submit reviews within three months after the set deadline.

The average reviewer responded to their invitation in 2.5 days and took 39.9 days to complete their review. The average number of reviews completed by individual reviewers during this time was 3.3. There were 776 late reviews, which were an average of 13.2 days late and 1,110 early reviews, which were an average of 11.1 days early. Reviewers recommended manuscripts to be accepted 10.6% of the time, to undergo major revisions 34.1% of the time, to undergo minor revisions 17.2% of the time, and to be rejected 38.1% of the time. JHE relied heavily on members of the Editorial Review Board to conduct reviews. This was very much a working Board, with the expectation of conducting 3–4 reviews per year.

Of the 4,121 manuscripts submitted to JHE during this timeframe and not withdrawn by their authors, 3,994 have received decisions. The remainder are either under review or returned to the authors for technical formatting or revision. Decisions are made at a variety of levels, and include rejections at the managing editor desk level (usually papers that have limited relevance to the field of higher education or do not meet the journal’s formatting or style specifications, for instance unsolicited essays and book reviews), rejections at the Editor in Chief level (subsequent review of the manuscript), rejections at the associate editor level (further review of the manuscript), rejections following the receipt of external peer reviews, and decisions to accept the manuscript following the receipt of external peer reviews. For our five-year sample, *The Journal of Higher Education* accepted 201 papers, rejected 2,475 papers at the managing editor level, rejected an additional 820 at the Editor in Chief level, rejected 131 at the associate editor level, and rejected 367 manuscripts following the receipt of external peer reviews. Accepted manuscripts were revised between one and four times, with an average number of revisions of 2.2. For the full timeframe in question, the average acceptance rate of *The Journal of Higher Education* was 5%, with the earlier years being slightly higher and the later ones being slightly lower. The range of acceptance rates was 4.4–6.5%, with lower acceptance rates largely driven by increased number of submissions to the journal.

### After Acceptance

Following a decision of accept, manuscripts enter the publication process with the journal’s publishing partner, Taylor & Francis. Over the period of several weeks, manuscripts undergo extensive copy editing and reference checking to ensure that anything not caught during the editorial process is resolved prior to publication. There are multiple checkpoints throughout this process, including the review of proofs—typeset and edited versions of the manuscript—by the editors of the journal and the author(s), as well as the authors’ response to a number of queries levied by the publication team. These queries range from anything as small as adding an institutional affiliation to locating the DOIs or stable weblinks of research papers referenced in the manuscript. Once the paper has exited the proofs stage, it enters the publication queue. *The Journal of Higher Education* is an online-first journal with a limited annual number of pages for hardcopy published editions of the journal and, as a result, newly published manuscripts appear online in advance of appearing in print. The journal had a 2020 impact factor of 3.108, a five-year impact factor of 3.769, and more than 135,000 downloads and views in the last year.

The average time to online publication for manuscripts submitted to and accepted by *The Journal of Higher Education* was 13 months, inclusive of the initial submission and processing time, the time required for external peer review, the time required for author revisions, any subsequent stages of additional peer review, and the production timeline. The production process is the least variable section of that timeline, and it takes between three to five weeks for authors to receive proofs from our publication team, approve those proofs, and finally have their manuscript published online.

Additionally, *The Journal of Higher Education* publishes manuscripts in print. The journal is budgeted a set number of volumes per year and a set number of pages across those volumes, which allows us to publish about 36 and 40 manuscripts each year, depending on the length of those manuscripts. Within the time frame discussed in this study, *The Journal of Higher Education* published an average of 37 manuscripts each year. Due to the constraints associated with print publishing, specifically page limits, the average time for manuscripts submitted to and accepted by *The Journal of Higher Education* to reach print was 16 months.

In addition to reviewing the publication process and timeline for manuscripts submitted to *The Journal of Higher* Education, we also reviewed the content of those manuscripts. In the next section, we take a closer look at these manuscripts*,* specifically focusing on the method utilized, submission region, and the topics addressed in the 4,121 manuscripts we reviewed over the course of the last five years.

## A Deeper Look at *The Journal of Higher Education’s* Submissions and Publications

Many of the preexisting studies of higher education research have focused on methodological, geographic, topical, and gender trends and preferences. Sharing the same interest of providing a more complete understanding of publishing within the study of higher education, we examined manuscripts submitted to JHE between 2017 and early 2022 to determine whether or not there are any statistically significant trends or preferences. Overall, our five-year sample of submissions included 3,994 manuscripts that have been rendered a decision. Of these 3,994 manuscripts, 201 were accepted for publication.

### Methodological Preferences

To test whether or not there is a preference for manuscripts that utilize quantitative methodologies*,* we first manually reviewed and coded each manuscript for methodological approach, either quantitative or qualitative, based on its title and abstract. When a method could not be identified from the title or the abstract, the manuscript was reviewed more closely to identify the method. Mixed methods manuscripts were submitted to the journal during this period but made up only a small fraction of the overall sample (less than 2%). So, they were coded by their primary method (i.e., a manuscript that claimed to have utilized a mixed methods study but was almost entirely qualitative and only conducted descriptive statistics was coded as having a qualitative approach).

We found that 64% of manuscripts submitted (*n* = 2,555) utilized a qualitative methodological approach, whereas 36% utilized a quantitative one (*n* = 1,439). Similarly, with respect to manuscripts that were accepted for publication, 58% (*n* = 116) utilized a qualitative methodological approach, whereas 42% (*n* = 85) utilized a quantitative one. Since there is a discrepancy within methodological approach between the percentage of manuscripts submitted and accepted, we conducted a two-sample t-test analysis of the publication rate of manuscripts by their methodological approach. We found that the acceptance rate for quantitative studies (5.9%) is significantly higher than that for qualitative studies (4.5%); t = 1.82 (*p* < 0.05). In short, although a larger number of qualitative studies were submitted and accepted, quantitative studies were accepted at a significantly higher rate than qualitative studies.

### Geographic Preferences

To test whether or not there is a preference for manuscripts submitted by authors who reside in the United States*,* we first manually reviewed and coded the submission country for each manuscript. Although JHE values the scholarship of colleagues working outside the U.S., the journal focuses on the U.S. context so studies conducted outside this context must make clear the relevance of the findings to U.S. higher education. Overall, 45% (*n* = 1,802) of manuscripts were submitted from authors in the United States. The other 55% (*n* = 2,192) were submitted from outside the U.S., representing 113 unique countries. In the interest of analytical clarity, we collapsed the submission location variable to a binary indicator of whether the paper was submitted from an author in the United States or outside of it to assess whether geographic origin mattered.

With respect to the papers that were accepted for publication, 95% (*n* = 191) were submitted by authors who resided in the United States, whereas 5% (*n* = 10) were submitted by authors residing in another country. A two-sample t-test analysis of publication rate by authors’ location shows that manuscripts submitted by those residing in the U.S. have a statistically significant higher likelihood of being published (10.59%) compared to their counterparts (0.4%) who reside in another country; t = -13.71 (*p* < 0.001).

### Institutional Preferences

Lastly, we considered whether published manuscripts were disproportionally authored more by those who were affiliated with a small group of institutions. Given that manuscripts were submitted by authors affiliated with 2,468 unique institutions and that no single institution represented more than 4% of accepted manuscripts (The University of Michigan), we did not conduct a more comprehensive analysis of this issue.

### Topical Preferences

To test whether or not there is a preference for manuscripts that address certain broad topical areas, we conducted structural topic modeling. Structural topic modeling allows researchers to assess the language located within a corpus of text documents and identify prominent themes and similarities across textual data, then assess the relationships between these themes and a key covariate (Roberts et al., 2014, 2018). For the purposes of this study, we assessed our sample of 3,994 manuscripts to identify key themes based on manuscripts’ titles then assessed the relationship of those themes with our key covariate of interest, namely whether or not the manuscript was published.

Our analysis began with a careful review of the dataset, informed partially by our experiences handling these manuscripts from submission to decision. We also manually reread each manuscript’s title again prior to structural topic modeling. The dataset of 3,994 manuscripts (documents, *d,* were specified as the paper’s title) had an overall vocabulary *v* (all words included in the dataset). We prepared the corpus by removing punctuation, links, numbers, and stopwords, which provide limited nuance and insight into the content of the text documents (i.e., words like and & the, which are semantically useful for constructing titles but less useful for understanding the relationships between words like state & funding, Benoit et al., 2018; Roberts et al., 2018). After preparing the corpus of text documents, we trimmed the dataset to include the 20% most reoccurring facets across the documents. While Roberts and colleagues (2018) recommend trimming the dataset to a smaller percentage of documents (5% or 10%), comparison between the 5% and 20% models indicates that the 20% model is a better fit for this data in terms of semantic coherence and topic specificity. This allowed us to identify a broader range of topics, important given the broad range of papers submitted to the journal, and limited noise in the data. After trimming, we removed empty documents (i.e., papers whose titles became shells after their contents were removed through our data preparation steps), which removed papers from consideration in which their titles offered no substantive information regarding the paper’s contents.

Following the completion of these data preparation steps, we ran a K estimation to determine the optimal number of topics with which to run our models. This step is the functional equivalent of deciding you have reached saturation with respect to qualitatively coding data or engaging in data reduction such as merging semantically similar codes. Topic number optimization is extremely necessary for structural topic modeling as, though the models utilize the data fed to them by the researcher, it is crucial to assess the desired range of topics to try to fit onto the data (i.e., for a smaller sample of text documents on a specific topic such as papers assigned for a class that share an assignment prompt, a lower number of topics may be more coherent than a larger number). We used the stm R package’s built-in feature to estimate this optimal number of topics (Roberts et al., 2018) and selected a topic number K by minimizing topic redundancy, maximizing semantic coherence of the output topics, and emphasizing topic uniqueness (i.e., merging topics like State Funding and State Allocations) to ensure a broad range of topics that had minimal overlap (Grimmer, 2010; Grimmer & Stewart, 2013). We estimated our optimum K value on Ks of 5, 10, 15, 20, 25, 30, 35, 40, 45, 50, 55, 60, 65, 70, and 75 and reviewed the held-out likelihood, residuals, semantic coherence, and lower bound of each model prior to identifying 25 topics as the optimal K value for our model.

After identifying our optimal K value, we employed a series of logistic-normal generalized linear models (Eq. 1) based on individual covariates X_d_ (decision rendered) over 75 iterations (Roberts et al., 2018).

1 $$\left.\mathrm{\theta d}\right|{\mathrm{X}}_{\mathrm{d\gamma }}, \sum \sim \mathrm{LogisticNormal}(\upmu ={\mathrm{X}}_{\mathrm{d}\gamma },\sum \hspace{0.17em}$$

where X_d_ is a 1-by-p vector, γ is a p-by-K − 1 matrix of coefficients and Σ is a K − 1 by K − 1 covariance matrix. This generated a document-specific topic distribution covaried by decision rendered.

We ran two topic models, the first which did not include the covariate and the second which did, to ensure that the topics did not materially change with the introduction of a covariate. They did not. After finalizing our model (*n* = 3,994), we qualitatively coded the 25 statistically prevalent topics (*p* < 0.001) and reviewed our codes together to ensure intercoder reliability for each topic (α = 1.00, complete agreement). We then assigned each topic a label based on our qualitative evaluation of the documents assigned to each topic (i.e., does a topic of ‘State Funding’ make sense for a handful of manuscripts about diversity measures and academic outcomes?).

After reviewing the topics and assigning labels, we removed 7 of 25 topics from our analyses due to limited semantic coherence (a string of words that did not make sense and documents that did not clarify what the topic might be) and retained 18 topics. These topics included: Indian Higher Education, COVID-19, Academic Success, Online Learning, Community Colleges and Transfer Students, Campus Climate, Pedagogy, International Students, Case Studies, STEM Students, Postgraduate Outcomes, Retention, Diversity, Equity, & Inclusion, Mental Health, Student Satisfaction, Funding & Policy, Career Trajectories, and Faculty Impact on Students. This broad range of identified topics suggests that there is significant topical diversity among the manuscripts submitted to *The Journal of Higher Education*.

With respect to the relationship between topic and publication, a handful of topics were significantly associated with one decision or the other. Manuscripts regarding online learning, which were submitted in large volume following the onset of the COVID-19 pandemic, were associated with higher rates of rejection (β = -0.03, *p* < 0.01). Manuscripts regarding community college students and the transfer student experience were associated with higher rates of acceptance (β = 0.05, *p* < 0.001). Likewise, manuscripts regarding diversity, equity, and inclusion efforts on college campuses were also associated with higher rates of acceptance (β = 0.032, *p* < 0.01). While all other topics listed above were statistically prevalent within the sample (i.e., each topic was submitted to the journal regularly), no other topics had a statistically significant relationship with either a positive or negative publication decision.

## Conclusion

Given the importance of publishing to academic life (Wilkins & Huisman, 2015), we drew from our experiences and insights as former Chief and Managing Editors of *The Journal of Higher Education* to shed light on the intricacies of the publication process and current trends within our field of study. Specifically, we described key stages of the review process and examined whether JHE leaned in favor of certain preferences during the course of a five-year period (2017–2022). Although JHE is not the only research journal that covers higher education research, it is the oldest and most-cited one among its peers (Earp, 2010). Because we had access to a more comprehensive sample of manuscripts than previous studies concerning publication of higher education research (Hutchinson & Lovell, 2004; Johnson et al., 2016; Wells et al., 2015; Williams et al., 2018), we were able to address a major limitation of those studies. That is, previous studies focused exclusively on published articles, whereas we also considered articles that were submitted but not offered publication.

Compared to other studies that have found that there was a preference for quantitative methodological approaches in top-tier higher education journals such as JHE (Johnson et al., 2016), our results only partially confirmed this preference. Although a larger number of qualitative studies were submitted and accepted, we also found that quantitative studies were accepted at a higher rate. Likewise, compared to other studies that have found that there was a preference for authors who reside in the U.S. (Fitzgerald & Jiang, 2020; Mason et al., 2021), our results also confirmed this preference. We found that although many manuscripts were submitted from outside of the U.S., only a few were accepted for publication. That authors who reside in the U.S. have significantly better odds of publishing in JHE than their international counterparts may be due to the narrow aim and scope of JHE, which prioritizes research that inform the US higher education context. Although we did not examine whether this effect was driven by focus of study, the pattern that we observed from our initial screening of manuscripts submitted from outside the U.S. was that nearly all of those spoke mainly to their own national context. Still, the imbalance is striking.

Lastly, our results from the structural topic modeling analyses show that research addressing topics about diversity, equity, and inclusion, as well as community college and transfer student success is associated with higher rates of acceptance. By contrast, studies that address challenges associated with online learning are associated with lower rates of acceptance. Despite these topical preferences, which were rather modest in effect, we found that a wide range of topics were addressed in manuscripts both at the submission and publication stages.

We acknowledge that there are limitations with our study. One limitation of structural topical modeling is the compression of nuance inherent to the approach. Put plainly, an explicitly qualitative approach (i.e., manually coding) may have resulted in different topics identified within the dataset though we would not have been able to statistically model these topics against their decisions as we were able to do with STM. With respect to ensuring that our structural topic models were as thorough as they could be, we painstakingly reread the titles and abstracts of all papers within the dataset and discussed the prevalent topics identified by the structural topic model in line with this manual review. Further, we isolated the most prevalent topics described by the model, collapsed other analytically redundant topics, and carefully reviewed the documents associated with each topic (i.e., the titles and abstracts of the papers that the model identified as representing each title) and verified that there was clear alignment between the topics and the origin content. We also chose not to disaggregate our decision analyses by rejection type (i.e., we could have explored whether a particular methodological type was desk rejected at a higher rate, but did not). It is doubtful given the size of the sample and the split between rejections and acceptances that this would have resulted in any statistically significant difference, but future work could more narrowly explore these topics. Similarly, future research could replicate these analyses using the submission data of multiple journals. Because our results were based on a five-year period associated with only one journal, we only hint at broader trends regarding publishing within the study of higher education. Still, our findings mostly confirm those from previous studies suggesting that journals prefer certain methodological approaches, geographic location, and topical areas.

At the same time, we are seeing signs of shifting trends. For example, more manuscripts utilizing a qualitative approach are reaching publication and research addressing access, equity, diversity, and inclusion has received more attention. Perhaps there are implications here for steering graduate education and for maximizing one’s chances for publication but because our findings are not causally based, we would caution against using them for those purposes. For us, our findings provide a snapshot of our field of study more than they suggest where we might be headed. Moving forward, we do not expect a journal’s review process and preferences to remain static but change albeit slowly in ways that reflect better the interests of their contributors and audience. Subsequently, we do not expect our findings from this study to be lasting features of our journals or our field of study. In this sense, research journals should not be preoccupied with signaling to researchers what they should be studying and how to study it, but instead should focus primarily on showcasing the most relevant and rigorous research per their scope and aim.
